# Weekly response assessment of involved lymph nodes to radiotherapy using diffusion-weighted MRI in oropharynx squamous cell carcinoma

**DOI:** 10.1118/1.4937791

**Published:** 2015-12-22

**Authors:** Neelam Tyagi, Nadeem Riaz, Margie Hunt, Kenneth Wengler, Vaios Hatzoglou, Robert Young, James Mechalakos, Nancy Lee

**Affiliations:** Department of Medical Physics, Memorial Sloan-Kettering Cancer Center, 1275 York Avenue, New York, New York 10065; Department of Radiation Oncology, Memorial Sloan-Kettering Cancer Center, 1275 York Avenue, New York, New York 10065; Department of Medical Physics, Memorial Sloan-Kettering Cancer Center, 1275 York Avenue, New York, New York 10065; Department of Radiology, Memorial Sloan-Kettering Cancer Center, 1275 York Avenue, New York, New York 10065; Department of Medical Physics, Memorial Sloan-Kettering Cancer Center, 1275 York Avenue, New York, New York 10065; Department of Radiation Oncology, Memorial Sloan-Kettering Cancer Center, 1275 York Avenue, New York, New York 10065

**Keywords:** diffusion-weighted MRI, oropharynx, adaptive re-planning

## Abstract

**Purpose:**

Patients with cancers of oropharynx have a favorable prognosis and are an ideal candidate for adaptive therapy. A replan to improve coverage or escalate/de-escalate dose based on morphological information alone may not be adequate as the grossly involved lymph nodes (LNs) of a subset of these patients tend to become cystic and often do not regress. Functional adaptation may be a better approach when considering replanning for these patients. The purpose of this study was to evaluate the weekly trends in treatment related morphological and physiological changes for these LNs using diffusion-weighted MRI (DW-MRI) and evaluate its implications for adaptive replanning.

**Methods:**

Ten patients with histologically proven oropharynx HNSCC undergoing concurrent chemoradiation were analyzed in this study. MR imaging protocol included axial T1w, T2w, and DW-MRI using a 3 T Philips MR scanner. The patients were scanned weekly in radiation treatment planning position using a 16 element phased-array anterior coil and a 44 element posterior coil. A total of 65 DWI and T2w scans were analyzed. DWI was performed using an optimized single-shot echo planar imaging sequence (TR/TE = 5000/65 ms, slice thickness = 5 mm; slices = 28; *b* values = 0 and 800 s/mm^2^). Quantification of the DW-MRI images was performed by calculating the apparent diffusion coefficient (ADC). T2w and DWI scans were imported into the Eclipse treatment planning system and gross tumor volumes (GTVs) corresponding to grossly involved LNs were contoured on each axial slice by physician experts. An attempt was made to remove any cystic or necrotic components so that the ADC analysis was of viable tumor only. A pixel-by-pixel fit of signal intensities within the GTVs was performed assuming monoexponential behavior. From each GTV histogram mean, median, standard deviation, skewness, and kurtosis were calculated. Absolute and percent change in weekly ADC histogram parameters and percent change in T2w GTV were also calculated.

**Results:**

For all nodes, an immediate change in ADC was observed during first 2–3 weeks after which ADC values either continued to increase or plateaued. A few nodal volumes had a slightly decreased ADC value during later weeks. Percent increase in median ADC from weeks 1 to 6 with respect to baseline was 14%, 25%, 41%, 42%, 45%, and 58%. The corresponding change in median T2 volumes was 8%, 10%, 16%, 22%, 40%, and 42%, respectively. The ADC distribution of the viable tumors was initially highly kurtotic; however, the kurtosis decreased as treatment progressed. The ADC distribution also showed a higher degree of skewness in the first 2 weeks, progressively becoming less skewed as treatment progressed so as to slowly approach a more symmetric distribution.

**Conclusions:**

Physiological changes in LNs represented by changes in ADC evaluated using DW-MRI are evident sooner than the morphological changes calculated from T2w MRI. The decisions for adaptive replanning may need to be individualized and should be based primarily on tumor functional information. The authors’ data also suggest that for many patients, week 3 maybe the optimal time to intervene and replan. Larger studies are needed to confirm their findings.

## 1. INTRODUCTION

Oropharynx squamous cell carcinoma (OSCC) associated with human papillomavirus (HPV) has a favorable prognosis. It is well established that HPV-positive patients are more responsive to chemotherapy and radiation therapy (RT) relative to HPV-negative patients,^1^ making them ideal candidates for adaptive therapy. An adaptive replan to improve the coverage or escalate/de-escalate dose can be beneficial to this population in further reducing normal tissue complications. Multiple groups are looking at prospective de-escalation protocols that favor deintensification of therapy. For instance, the Eastern Cooperative Oncology Group (ECOG) Phase II study (E1308) that evaluated whether response to platinum-based induction chemotherapy could be used to select patients who can safely receive a lower dose of intensity modulated radiation therapy (54 instead of 74 Gy). Two other clinical trials (NCT1088802/J0988 and NCT01221753) are exploring other radiation deintensification protocols with concomitant chemotherapy in favorable subset of HPV-associated OSCC.

Because of current effort towards deintensification of therapy, a better understanding of how tumors regress during treatment particularly for rapidly responding malignancies such as HPV-positive OSCC is essential in determining the best time-point for replanning. A replan to improve coverage or escalate/de-escalate dose based on morphological information alone may not be adequate as the grossly involved lymph nodes (LNs) of HPV-positive oropharynx patients tend to become cystic and often do not regress. Functional adaptation may be a better approach when considering replanning for these patients. Existing techniques to monitor during-treatment tumor-related changes are based on morphological information derived from CT, CBCT, and anatomical T2w MRI.^2–5^ Although changes in normal structure volumes may be estimated with fair certainty from such longitudinal studies, the accuracy in assessing tumor volume change based on midtreatment CT or CBCT may not be sufficient due to the poor soft tissue contrast. Even high quality imaging, such as T2w MRI, may not be sufficient as it only provides volumetric, and not functional, information about the tumor.^6^ Rather, such decisions may require information regarding the physiological and functional characteristics of the tumor. Although PET has been used to extract this information, the frequency at which PET can be acquired is limited,^7,8^ compromising our ability to determine the optimal time for replanning from serial imaging studies.

One noninvasive technique that can be acquired periodically during treatment to provide physiological information about the tumor is diffusion-weighted MRI (DW-MRI). DW-MRI exploits the random motion of water molecules to detect relatively small changes in tissue structure at the cellular level. The extent of tissue cellularity and the presence of an intact cell membrane affect the impedance of water molecule diffusion which can then be quantitatively assessed using the apparent diffusion coefficient (ADC) value. DW-MRI is quick and easy to perform and provides an opportunity to quantitatively assess therapeutic-induced changes in solid tumors over time.^9–11^ Furthermore, incorporating DW-MRI allows both conventional morphologic and physiologic assessments to be made during the same examination.

The purpose of this study is to evaluate weekly trends in morphological and physiological changes based on T2w and DW-MRI, respectively, for the gross lymph node tumor volumes and evaluate its implications for adaptive replanning, particularly the time-point at which adaptive re-planning will be most effective. A future goal of this study is to identify, based on individualized weekly trends, patients who will need dose escalation or can be safely dose de-escalated.

## 2. METHODS AND MATERIALS

### 2.A. Patient selection and imaging protocol

Ten patients [8 men, 2 women; median age: 58 yr (range 51:61 yr)] with histologically proven oropharynx HNSCC undergoing concurrent chemoradiation were enrolled in a prospective IRB study (IRB No. 04-070) to undergo weekly MRI scans in radiation treatment position. Patient characteristics are described in Table I.

All patients received concurrent cisplatin except patients 4 and 6 who received a combination of carboplatin, cisplatin, and paclitaxel. Each patient had gross lymph node involvement and treatment planning was done according to institutional guidelines.^12,13^

Each nodal gross tumor volume (GTV) received a total prescription dose of 70 Gy in 35 days (2.0 Gy/fx). A total of 15 (1 nodal volume in 7, 2 nodal volumes in 2, and 4 in 1 patient, respectively) grossly involved lymph node volumes were followed longitudinally. Multiple GTVs on the same patient were analyzed separately. Routine pretreatment simulation imaging included PET-CT and multiparametric MRI in a 5-point thermoplastic mask. Additional weekly on-treatment anatomical and DW-MRI scans were also obtained as described below. A total of 65 DW-MRI and 65 T2w scans were analyzed in this study. The mean interval between simulation and the first week of treatment was 15 days. Median interval (in days) from RT start date to MRI scan during week 1, 2, 3, 4, 5, and 6 was 6, 13, 20, 27, 34, and 41, respectively.

**FIG. 1.**
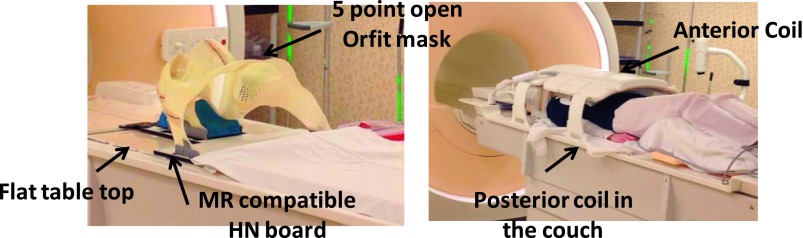
Head and neck MR simulation setup.

The imaging protocol included anatomical high resolution axial T1w, T2w, T2 fat saturated and DW-MRI using a 3 T Philips Ingenia scanner (Philips Medical System). The patients were scanned in radiation treatment planning position using a 16-element phased array anterior coil and a 44-element posterior coil. Figure 1 shows the patient setup for the MRI acquisition.

DW-MRI was performed using an optimized single-shot echo planar imaging sequence and two *b*-values (0 and 800 s/mm^2^) and single diffusion gradient direction (TR = 5000 ms, TE = 65 ms, flip angle = 90°, 128 × 128, slice thickness: 5 mm, number of slices = 28, four signal averages and bandwidth = 20 KHz/pixel) was used. Anatomical scans included a high resolution axial, 3D T2-weighted turbo spin-echo sequence (TR/TE = 1800/250 ms, slice thickness = 1 mm and in-plane resolution of 1 mm^2^, FA = 90°, number of slices = 300) and 2D T1-weighted sequence (TR/TE = 760/18 ms, slice thickness = 2 mm and in-plane resolution of 1 mm^2^, FA = 90°). T2 fat saturated (TR/TE = 3000/80 ms, slice thickness = 5 mm and in-plane resolution of 1 mm^2^, FA = 90°, number of slices = 28) sequence matching the DW-MRI acquisition was also acquired. In addition to DW-MRI, a dynamic contrast enhanced scan was also obtained for each patient during the pretreatment scan procedure to confirm localization of malignant tissue as well as any cystic and/or necrotic volume. Gadolinium contrast enhanced scans were not acquired during the weekly scan procedures. Changes in morphological and physiological information were derived from weekly T2w MRI and DW-MRI only.

### 2.B. Diffusion-weighted imaging analysis

With diffusion imaging, the signal typically declines exponentially as a function of *b* values (or diffusion weighting). Quantification of this signal loss is performed by calculating the ADC from the generic formula

$$S\left(b\right)=S\left(0\right)e^{-b*\text{ADC}},$$ (1)

where *S*(*b*) is the signal intensity measured using a *b* value “*b*” and *S*(0) is the signal intensity for *b* = 0 s/mm^2^.

T2w, b0, and b800 DW-MRI scans were imported into our research Eclipse (Varian Medical Systems) treatment planning system (TPS). Manual and deformable registrations were used to register weekly DW-MRI and T2 to the corresponding pretreatment scans, and manually edited if needed. Grossly involved lymph nodes were then contoured on each axial slice of each weekly T2w and DW-MR scan by expert radiation oncologists (NL and NR) and radiologists with 10 yr (HY) and 15 yr (RY) of experience. Prior to analyzing the DW-MRI to determine ADC for each node, an attempt was made to remove any cystic or necrotic components so that the analysis was of viable tumor only. This was done by subtracting the cystic/necrotic region of interest (ROIs) from the entire GTV ROIs using Boolean operators in the TPS. For each node, the presence of cystic or necrotic components was determined from the T1w, T2w, T1 postcontrast and both the low and high *b* value DW-MRI scans. The limited or no uptake on pretreatment postcontrast T1 scan was used to confirm the presence and location of necrotic volumes identified on the pretreatment DW-MRI; however, for the weekly imaging, contrast enhanced studies were unavailable, and therefore, the only confirmation that was possible was of the DW-MRI-defined cystic components using T1w and T2w images. Other studies have made use of higher *b* value scans in delineating necrotic volumes.^14^ Segmentation reproducibility of viable tumor was tested by evaluating the weekly trends in ADC reproducibility by the two radiologists on four representative cases. For volumetric analysis on weekly T2w images, entire GTV including cystic and necrotic component was analyzed to adhere with the current clinical practice.

**FIG. 2.**
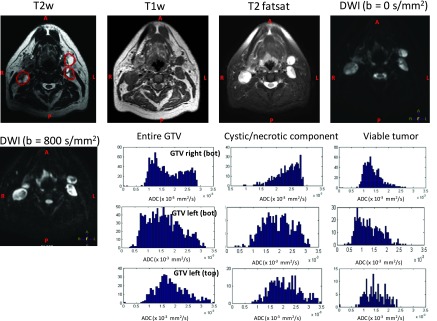
GTV nodal volume shown on T2w, T1w, T2 fatsat, DWI (*b* = 0 and 800) of a representative patient with 4 nodal volumes (pat 6). The histograms represent the ADC distribution for the entire tumor (left), the cystic/necrotic component of the tumor (middle), the viable tumor (right) for right (GTV 8), and left GTV nodal volumes (bottom or GTV 9 and top or GTV 10) for this representative patient.

Figure 2 shows the T1w, T2w, T2 fat saturation and DW-MRI (*b* = 0 s/mm^2^, *b* = 800 s/mm^2^) images of a representative patient with multiple nodal volumes (Patient No. 6). The figure shows three gross lymph node volumes of the patient drawn on the T2w MRI. Also shown are the ADC histogram distributions of left top, left bottom, and right bottom nodal volumes. The histograms represent the ADC distribution of the entire GTV, cystic/necrotic component, and the viable tumor only.

### 2.C. Statistical analysis

For each patient, the 3D GTV ROIs were exported as dicom RT structures from the TPS and read into a matlab (MathWorks Inc, MA, USA) program. A pixel-by-pixel fit based on monoexponential behavior was calculated using Eq. (1) and histograms were generated. From each GTV histogram, the following descriptive parameters were calculated: mean, median, standard deviation, percentile (10%, 25%, 75%, and 90%), kurtosis, and skewness. These parameters represent the first-order statistical properties of the image. Mean and standard deviations represent average and dispersion of the histogram, respectively. A percentile represents the value below which a percentage of observations are calculated. Kurtosis reflects the peakedness of the distribution and is a measure of the shape of the probability distribution. A histogram with positive kurtosis has a higher probability of values being distributed near the mean value than does a normally distributed histogram. A negative kurtosis indicates a histogram that has more pronounced tails than does a normally distributed histogram and indicates a higher probability of extreme intensity values than that for a normally distributed histogram. Skewness represents a measure of asymmetry of the probability distribution. A positive skew indicates a longer tail to the right of the histogram, and a negative skew indicates a longer tail to the left of the histogram.

Absolute and percent change in weekly ADC (×10^−3^ mm^2^/s) histogram parameters of the viable tumors and change in weekly T2 GTV nodal volumes (in cm^3^) were calculated. Analysis for all 15 GTVs was combined into population statistics using box plot diagrams. Population median, skewness, and kurtosis for weekly ADC trends as well as percent change in median ADC and T2 GTV nodal volumes with respect to pretreatment scan were also generated. Correlation between weekly changes in ADC value of viable tumor and changes in GTV on T2w MRI was investigated by calculating Pearson correlation coefficient within the stata (STATA Corp., LP, TX, USA) statistical package. A *p* value less that 0.05 will be considered statistically significant.

## 3. RESULTS

**FIG. 3.**
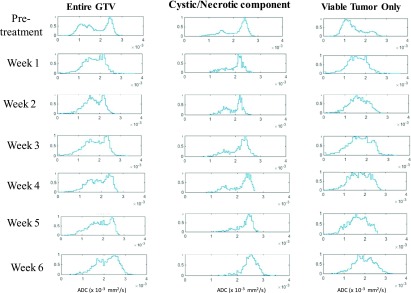
Weekly trend in histogram distribution of the entire tumor (left), the cystic/necrotic component of the tumor (middle), and the viable tumor (right) for a patient with large cystic/necrotic component (pat 10, GTV 15). The entire GTV represents a bimodal distribution which makes it difficult to evaluate the trend in weekly response assessment. Separating the tumor volume into viable tumor only and cystic/necrotic components gives a clear indication of true response assessment.

Figure 3 shows the weekly histogram trend of the entire GTV, cystic/necrotic component, and the viable tumor only for a patient with a large cystic/necrotic node before treatment that persisted throughout the treatment (Patient No. 10, GTV 15). The entire GTV shows a bimodal distribution, whereas after removing the cystic component, represented by higher ADC values, a unimodal distribution for the viable tumor remains. For weekly trend analysis, only the viable GTV tumor volume was investigated.

Segmentation reproducibility of viable tumor was tested by evaluating the weekly trends in ADC reproducibility by the two radiologists. The interobserver agreement for median ADC values calculated based on intraclass correlation coefficient (ICC) was 0.97 [95% CI = 0.937 0.987] indicating a good agreement in the median ADC values and the weekly trends in ADC.

### 3.A. Individual trend in GTV LN based on DW-MRI and T2w-MRI

**FIG. 4.**
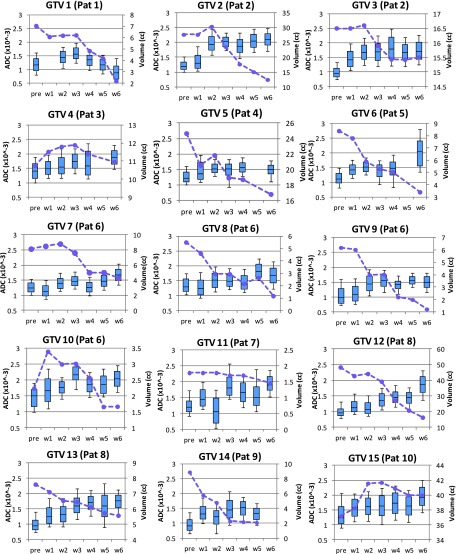
Trends in ADC histograms (shown as box plots) of the viable tumor (left vertical axis) and T2w tumor volume shown as dashed line (right vertical axis) for the 15 GTV nodal volumes under study.

Figure 4 shows the weekly ADC trends for viable tumor only on the left vertical axis and T2w tumor volume on the right vertical axis for all 15 GTVs individually during the course of chemo-RT. Histograms trend in ADC values of the nodal volumes is represented by a box plot with the box representing 25th and 75th percentile. The horizontal line in the box is the median value. The weekly trend in T2w tumor (in cm^3^) is represented by dashed lines. The median pretreatment ADC values were 1.19 × 10^−3^ mm^2^/s (range: 0.92 × 10^−3^ to 1.51 × 10^−3^ mm^2^/s) and median tumor volume was 8.4 cm^3^ (range: 2–48 cm^3^). For all nodes, an immediate change in ADC is observed during first 2–3 weeks after which the ADC value either continues to increase (GTV 4, 6, 7, 10, 12, 13, and 15) or plateaus (GTV 2, 3, 5, 8, 9, 11, and 14). The one exception was nodal volume 1 for which ADC continued to decrease after week 3. Although GTV 3, 5, 8, and 14 plateau after week 2, all four GTVs also show a slight decreased ADC during later weeks.

**FIG. 5.**
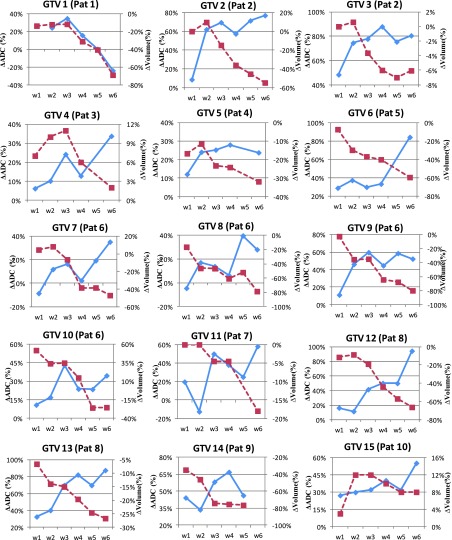
Percent change in median ADC values of the viable tumor (left vertical axis and solid lines) and T2w tumor volume (right vertical axis and dashed lines) for the fifteen GTV nodal volumes.

The tumor volumes on T2w MRI continued to decrease during RT with the largest change observed in GTV 12 (50–15 cm^3^) and lowest observed in GTV 3 (16.5–15.5 cm^3^). GTV 4, 10, and 15 became increasingly cystic in the earlier weeks, making the tumor nodal volume bigger as well. Some of the fluctuations seen in other GTV occurred because either the tumor volume increased due to more cystic nature or the cyst began to separate with chemo-RT making the tumor boundary slightly diffuse on the T2w MRI. Six out of 15 GTVs either did not show any change in tumor volume or had a slight increase in tumor volume in early weeks. The tumor volumes did not generally begin to shrink until week 3. We looked at percent change in median ADC and tumor volume with respect to pretreatment values. The percent changes for all 15 GTVs are shown in Fig. 5. GTV 5 showed a very small change in percent median ADC that reached plateau immediately and also shows a slight drop in median ADC at the end of the treatment.

### 3.B. Response assessment

**FIG. 6.**
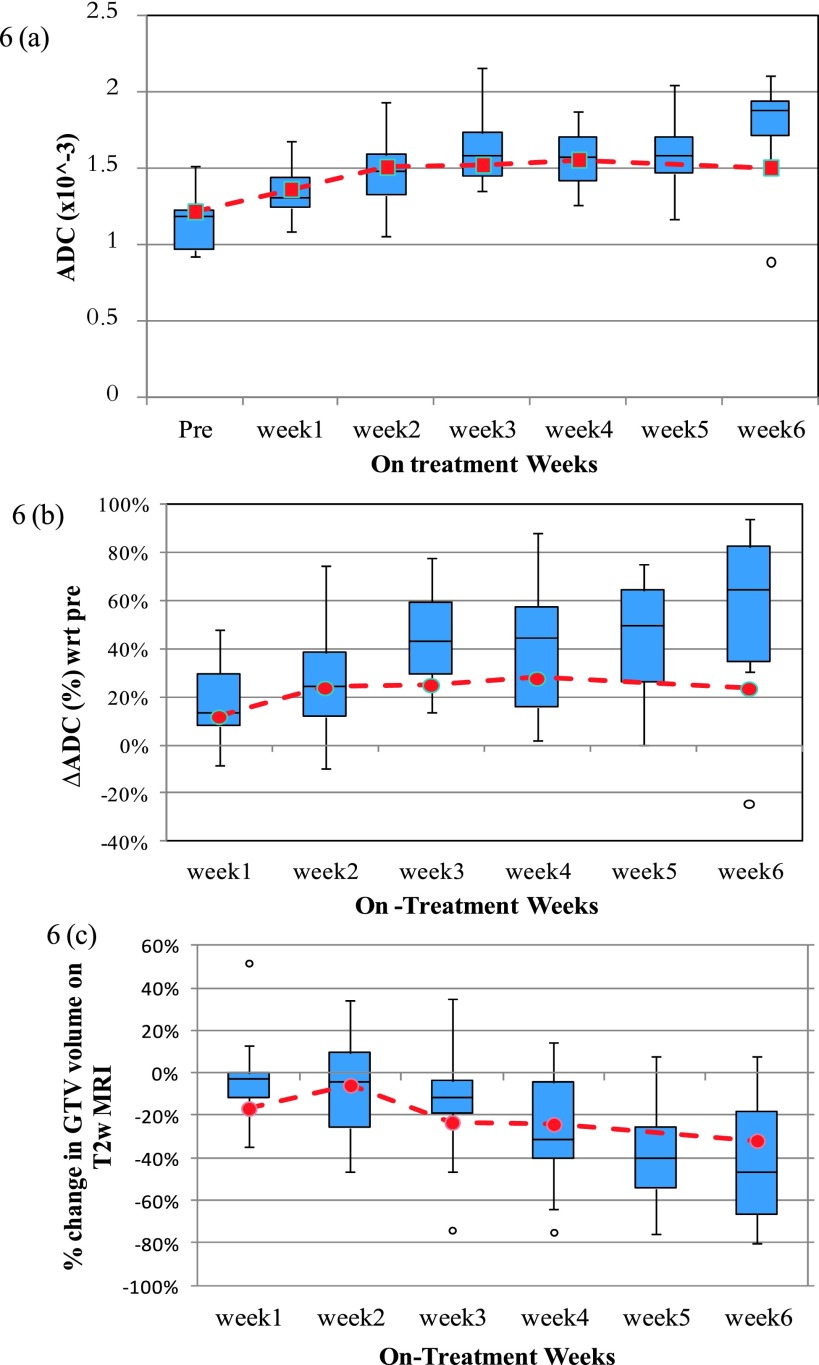
Population trend in (a) median ADC of the viable tumor, (b) percent change in median ADC of the viable tumor from the pretreatment value, and (c) percent change in T2w GTV with respect to the pretreatment volume on T2w MRI. The box plots represent the population trend for complete responders (total = 14 nodal volumes). The red dashed line represents the trend for a partial responder.

The effect of change in ADC due to concurrent chemo-RT on the patient response was assessed based on three months post-RT ^18^FDG-PET. Out of 15 GTV nodal volumes, 3 volumes had persistent disease at three months post-RT ^18^FDG-PET [GTV 3 (Pat 2), GTV 5 (Pat 4), and GTV 8 (Pat 6)] and were grouped as slow responders. Out of these three GTVs, two of them (GTV 3 and 8) were negative for disease after neck dissection. For GTV 3, the median ADC for the viable tumor was lower (0.999 × 10^−3^ mm^2^/s) before RT and plateaued after week 3. However, the tumor volume only changed from 16.5 to 15.5 cm^3^ (∼6% decrease) as the cystic/necrotic component kept growing. Necrotic areas are often associated with hypoxic areas that are resistant to radiotherapy and may indicate poor treatment outcome. GTV 8 showed little or no change in ADC until week 4 of chemo-RT. ADC skewness continued to decrease while kurtosis remained unchanged. GTV 5 had residual cancer after neck dissection and was a HPV-negative patient. This patient was grouped as a partial responder. The percent change in ADC response was small compared to other GTV. The ADC trend also showed a slight decrease in ADC at the end of the treatment (Fig. 6). All the remaining GTVs had no evidence of disease (NED) during follow up and remained NED 1 yr post-RT. They were grouped as complete responders. The exception to the ADC trend was GTV1 which was HPV-positive tumor and remained NED 1 yr post-RT.

Our patient population had only two HPV-negative patients (GTV 5 and 14). GTV 5 showed a very small change in percent median ADC that reached plateau immediately and also showed a slight drop in median ADC at the end of the treatment. The percent change during week 1–week 6 with respect to baseline scan for this tumor was 12%, 24%, 25%, 28%, 26%, and 24%, respectively. This patient also had persistent disease on PET after RT. The other HPV-negative patient (GTV 14) showed a trend similar to GTV 5 but had lower ADC value before chemo-RT and had a percent median ADC change of 44%, 34%, 58%, 67%, and 46%, respectively, during the first 5 weeks. During later weeks, GTV 14 behaved very much like a HPV-positive tumor. The lower pretreatment ADC value (0.962 × 10^−3^ mm^2^/s for GTV 14 vs 1.265 × 10^−3^ mm^2^/s for GTV 5) was very sensitive to chemotherapy and radiation and showed greater response as compared to GTV 5. Other studies have also shown that pretreatment ADC of complete responders was significantly lower than that from partial responders.^15^ This variability in our preliminary data for HPV-negative tumors may suggest the potential of weekly monitoring of ADC values in a larger dataset to evaluate treatment response.

### 3.C. Population trend in GTV LN based on DW-MRI and T2w-MRI

The population trend for median ADC and percent change in median ADC with respect to pretreatment values are shown in Figs. 6(a) and 6(b). The box plot in the figure shows population trend for complete responders (total = 14). The red dashed line shows the trend for the partial responder (GTV 5). The population trend shows a rapid increase in median ADC until week 2–3 as a result of chemo-RT. Beyond week 3, the ADC value reaches a plateau and then begins to increase for the complete responders at the very last week. For partial responder, the ADC value does not change much beyond week 3. The population trend in percent change in median ADC is more sensitive and shows a continuous increase in ADC of 60% from baseline value by week 6. For the partial responder, the %ADC change shows a very slow increase of 28% from baseline value by week 4 and then shows a slight decrease at the end. Population changes in the morphological nodal volumes analyzed on the T2w images are shown in Fig. 6(c). The percent change in GTV on T2w image is smaller for partial responder as compared to complete responders.

In comparison to percent change in median ADC, percent change in GTV does not change as rapidly during the first few weeks of chemo-RT but is more evident later in the treatment. Percent change in T2 volume does not reach 40% until about week 5 for complete responders. This implies that physiological changes in tumor evaluated using DW-MRI are evident sooner than the morphological changes in tumor volume calculated from T2w MRI. The majority of the nodal volumes (13 of 15) in this study are HPV-positive and it is known that these tumors tend to become cystic rather than shrink. Nonetheless, a nonsignificant, weak, negative correlation (*ρ* = − 0.105, *p* = 0.38) was found between changes in ADC of the viable tumor and changes in GTV on T2w MRI for the entire population. We also did not find any correlation between weekly changes in ADC values with weekly changes in GTV on T2w MRI suggesting that functional and anatomic changes represent independent response parameters. The general trend for percent change in ADC and percent change in T2 volume for the partial responder is not very different.

**FIG. 7.**
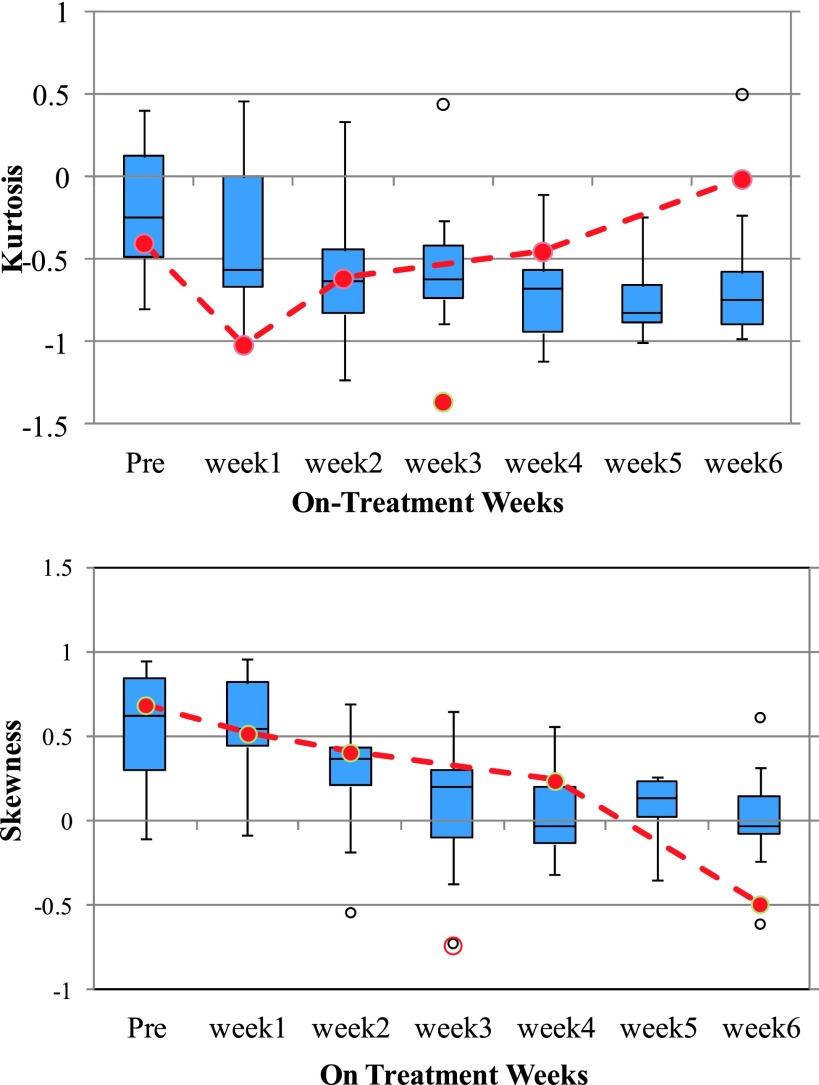
Population kurtosis and skewness of the ADC of the viable tumor only for the GTV nodal volumes. The box plots represent the population trend for complete responders (total = 14 nodal volumes). The red dashed line represents the trend for a partial responder.

A population trend of the weekly changes in kurtosis and skewness of the ADC histograms of the viable tumors is shown in Fig. 7. The ADC distribution of the tumors is initially Gaussian (as represented by kurtosis ∼0); however, the kurtosis decreases as treatment progresses for complete responders and is most evident from week 2 onward. For the partial responder, the kurtosis shows an increasing trend after week 1. Population skewness box plot shows that the histogram distribution shows a higher degree of skewness in the first 2 weeks, progressively becoming less skewed as treatment progresses so as to slowly approach a more symmetric distribution. For the partial responder, the skewness continues to decrease.

Eleven out of fifteen GTVs had cystic/necrotic component. In 3 GTVs (4, 10, 15), the cystic/necrotic component remained from the beginning till the end of the treatment. In 5 GTVs, the component developed after week 1 or week 2 into RT (2, 3, 5, 6, and 13) that completely resolved at the end of last fraction except GTV 3 that remained persistent with a necrotic volume of 5 cm^3^. The necrotic volume grew bigger in the beginning weeks for this GTV. In three patients, the cystic component resolved in the early weeks (8, 9, 11). Some of these volumes were scattered or diffused either in the beginning or at the end or throughout RT that made it slightly challenging to delineate them. The most accurate way to confirm these volumes is with the help of a contrast enhanced scan during the treatment which was not available.

## 4. DISCUSSION

DW-MRI has been shown to be an effective early biomarker for treatment outcome for both antivascular drugs as well as therapies that induce tumor cell apoptosis. Successful treatment is reflected by an increase in ADC values, and this finding has been noted for several anatomic sites including HNSCC.^16^ Tumors with a lower ADC value are more likely to have viable proliferative cells, which are sensitive to chemotherapy and radiotherapy. Conversely, the presence of inflammatory changes, necrosis, and fibrosis influences ADC value, which is correlated with interstitial water content and low cell density in histological samples.^17,18^

Oropharynx nodal volumes, especially HPV-positive nodes, become more cystic/necrotic over the course of RT. Necrotic/cystic tissues show high ADC values resulting from larger diffusion distances as a consequence of lost membrane integrity.^19^ A necrotic tissue may sometime be regarded as a cystic component even when contrast enhancement with gadolinium is performed. For our analysis, both components were lumped together and subtracted from the viable tumors. We used the ADC value of the viable tumor and avoided the cystic and necrotic areas to not affect the ADC value of the tumor. An early increase in ADC of the viable tumor was seen within week 2–week 3. This early increase in ADC is the result of an increase in the fractional volume and diffusion of molecules in the extracellular space that occurs with cell shrinkage and death, and movement of water from the intracellular to the extracellular space as a result of chemotherapy and radiation. After week 2 or 3, a plateau was reached, presumably because tumor microvasculature and tumor cell environment had been effectively destroyed by concurrent chemo-RT and diffusion could no longer increase.

Out of 15 GTV nodal volumes, 3 volumes had persistent disease at one month post-RT ^18^FDG-PET (GTV 3, GTV 5, and GTV 8) but only one volume was positive on pathology. For this partial responder, the percent change in ADC was less than 25% by week 3. The study by Vandecaveye *et al.* has also shown that a percent change in ADC of <25% in early weeks of RT resulted in poor response in their study group.^14^ Although there was only one partial responder in our group, the general trend agrees with the data published in the literature. Our data support the idea that it may be possible to predict outcome based on early changes in ADC trend. The decreasing ADC trend in skewness and kurtosis, while remaining positive, has been shown to be a potential indication of positive treatment response in other disease sites.^20^ Based on the ADC trend in median, skewness, and kurtosis, the slow responders could potentially be on a close watch. Even though a replan is no longer possible for these patients, post-RT intervention could be considered.

We found a negative correlation between changes in the ADC value of viable tumor and changes in GTV on T2w MRI. The correlation was not statistically significant implying that it is not possible to predict GTV shrinkage volume based on the ADC trend. Previous studies have also shown that changes in the two and four week ADC correlated with loco-regional control but not the volume changes.^14^ The implication of these data is that adaptive replanning decisions, particularly those involving dose escalation to viable tumor and/or de-escalation to cystic/necrotic regions, should rely as much or more on physiological changes than morphological change. A cystic volume either remains persistent throughout the treatment or gets absorbed during treatment either due to chemo-RT or due to lymphatics although there is no clear evidence to suggest the contributing factor. In some cases, the cystic or necrotic volume resolved at the end of the treatment which other authors have also observed.^14,21^

Our data suggest the importance of weekly diffusion imaging in adaptive replanning decisions as different patients behave differently. Our data based on physiological properties of the tumor indicate that week 3 may be the best time-point for replanning. Pretreatment and post-treatment imaging may not be effective and sufficient to predict clinical outcome. Rather serial changes in ADC may predict local control more accurately as also observed by other authors.^14,22^ For patients with decreasing ADC at the end of the RT treatment, serial imaging may enable us to plan for post-RT intervention. Serial imaging also enables us to follow the foci of cyst/necrosis that display high ADC values and are often associated with hypoxic areas resistant to radiotherapy. Currently, FMISO PET is the only modality adopted in the clinic that predicts hypoxia specific changes. Further studies are needed to confirm the correlation between FMISO and DWI specific hypoxia. Recently, with the introduction of high tesla MR simulators and MR-Linac systems in the radiation oncology clinics, it has become feasible to acquire periodic on-treatment multiparamteric MRIs that can incorporate both morphological and physiological information.

The viable tumor and cystic/necrotic volumes were drawn on pretreatment volumes based on postcontrast T1 since the gadolinium contrast was given only during simulation. For weekly diffusion images, the T1, T2, and high *b*-value images were used to draw viable and cystic/necrotic volume. We found that it was essential to separate viable tumor from necrotic component. The distribution would otherwise show a bimodal distribution either in the beginning of therapy or middle and end which made it difficult to analyze a trend based on the entire volume. The radiologists used their training and experience to delineate these structures but unless we use a threshold based segmentation method, there will be a few overlapping pixels in both regions. We could not find any appropriate threshold value in the literature to use for our segmentation methodology. Although our results show good reproducibility of ADC values between the two observers, the segmentation of viable and necrotic portion may be somewhat inaccurate based on our method.

The study has following limitations. Only ten patients were analyzed in this pilot study. Because of the low patient population, the standard deviation in our population DWI metrics is high. In addition, only two of fifteen tumors were HPV-negative. Future studies will therefore focus on analyzing the data on a larger patient population to improve statistical power. Many patients entered onto our protocol could not be analyzed due to excessive distortion in the DW-MRI images resulting from the fact that imaging was performed with patients in the treatment position. The presence of many tissue-air interfaces in the head and neck region makes DW-MRI very challenging to perform in the radiotherapy treatment position using an anterior–posterior coil combination. A dedicated neurovascular coil provides a much more homogeneous *B*_1_ distribution compared to a phased-array coil but most radiotherapy immobilization devices are not compatible with a dedicated diagnostic coil. We also observed significant susceptibility artifacts leading to distortion that prevented analysis of the primary tumors. The EPI gradient echo based technique used for diffusion imaging has inherent problems with susceptibility, especially in the presence of many air-tissue boundaries. Alternative acquisition schemes such as single-shot turbo-spin echo will be less affected by susceptibility artifacts and may be feasible in treatment position. We are currently exploring the use of such a sequence along with a combination of flex, anterior and posterior surface coils in treatment position for both anatomical and diffusion scans.

Another limitation of this study was the use of 2 *b*-values (0 and 800 s/mm^2^) which was chosen to prove the clinical feasibility of acquiring weekly diffusion images in treatment position. However, because of fewer *b*-values, we could not investigate the effect of intravoxel incoherent imaging (IVIM) in this longitudinal study. Data from our institution have suggested that primary tumors and metastatic nodes show significant perfusion fraction measured by IVIM.^23^ Since *b* = 0 s/mm^2^ is also used for IVIM modeling, our analysis may be overestimating the ADC values. We intend to incorporate the effect on IVIM on weekly response assessment in future studies.

## 5. CONCLUSION

There is significant variability in the temporal pattern of anatomic regression between patients, suggesting that individualized time points may be necessary for optimal adaptive planning. Cystic nodes do not change in volume significantly during the course of RT; however, physiological changes are apparent early on in the treatment. Our pilot study indicates that physiological changes represented by changes in ADC occur earlier than the change in volume. These data suggest that anatomic information alone may not be sufficient to assess tumor response that decisions for adaptive replanning may need to be individualized and based primarily on functional or physiological data. Our data also suggest that for many patients, weeks 3–4 may be the optimal time to intervene and replan. Larger studies are needed to confirm our findings.

## Figures and Tables

**TABLE I.** Patient specific parameters.

Patient No.	Site	Age at diagnosis	TN staging	HPV status	Number of nodal volumes
1	BOT	55	T2N2c	Positive	1
2	Tonsil	51	T2N2c	Positive	2
3	BOT	57	T3N2	Positive	1
4	Neck	58	TxN2	Negative	1
5	Tonsil	59	TxN2	Positive	1
6	BOT	59	T3N2	Positive	4
7	Unknown	61	TxN2c	Positive	1
8	Neck	58	T2N2c	Positive	2
9	BOT	56	T3N2	Negative	1
10	Tonsil	58	T2N3	Positive	1

Note: BOT = base of tongue.

## References

[1] K. K. Ang, J. Harris, R. Wheeler, R. Weber, D. I. Rosenthal, P. F. Nguyen-Tan, W. H. Westra, C. H. Chung, R. C. Jordan, C. Lu, H. Kim, R. Axelrod, C. C. Silverman, K. P. Redmond, and M. L. Gillison, “Human papillomavirus and survival of patients with oropharyngeal cancer,” N. Engl. J. Med. 363(1), 24–35 (2010). 10.1056/NEJMoa0912217 20530316 PMC2943767

[2] P. Castadot, J. A. Lee, X. Geets, and V. Gregoire, “Adaptive radiotherapy of head and neck cancer,” Semin. Radiat. Oncol. 20(2), 84–93 (2010). 10.1016/j.semradonc.2009.11.002 20219546

[3] D. L. Schwartz, A. S. Garden, J. Thomas, Y. Chen, Y. Zhang, J. Lewin, M. S. Chambers, and L. Dong, “Adaptive radiotherapy for head-and-neck cancer: Initial clinical outcomes from a prospective trial,” Int. J. Radiat. Oncol., Biol., Phys. 83(3), 986–993 (2012). 10.1016/j.ijrobp.2011.08.017 22138459 PMC4271827

[4] C. Veiga, J. McClelland, S. Moinuddin, A. Lourenco, K. Ricketts, J. Annkah, M. Modat, S. Ourselin, D. D’Souza, and G. Royle, “Toward adaptive radiotherapy for head and neck patients: Feasibility study on using CT-to-CBCT deformable registration for ‘dose of the day’ calculations,” Med. Phys. 41(3), 031703 (12pp.) (2014). 10.1118/1.4864240 24593707

[5] D. Yan and J. Liang, “Expected treatment dose construction and adaptive inverse planning optimization: Implementation for offline head and neck cancer adaptive radiotherapy,” Med. Phys. 40(2), 021719 (10pp.) (2013). 10.1118/1.4788659 23387742

[6] A. M. Chen, M. E. Daly, J. Cui, M. Mathai, S. Benedict, and J. A. Purdy, “Clinical outcomes among patients with head and neck cancer treated by intensity-modulated radiotherapy with and without adaptive replanning,” Head Neck 36(11), 1541–1546 (2014). 10.1002/hed.23477 23996502

[7] R. N. Moule, I. Kayani, T. Prior, C. Lemon, K. Goodchild, B. Sanghera, W. L. Wong, and M. I. Saunders, “Adaptive ^18^fluoro-2-deoxyglucose positron emission tomography/computed tomography-based target volume delineation in radiotherapy planning of head and neck cancer,” Clin. Oncol. 23(5), 364–371 (2011). 10.1016/j.clon.2010.11.001 21109410

[8] B. A. Hoeben, J. Bussink, E. G. Troost, W. J. Oyen, and J. H. Kaanders, “Molecular PET imaging for biology-guided adaptive radiotherapy of head and neck cancer,” Acta Oncol. 52(7), 1257–1271 (2013). 10.3109/0284186X.2013.812799 24003853

[9] D. A. Hamstra, T. L. Chenevert, B. A. Moffat, T. D. Johnson, C. R. Meyer, S. K. Mukherji, D. J. Quint, S. S. Gebarski, X. Fan, C. I. Tsien, T. S. Lawrence, L. Junck, A. Rehemtulla, and B. D. Ross, “Evaluation of the functional diffusion map as an early biomarker of time-to-progression and overall survival in high-grade glioma,” Proc. Natl. Acad. Sci. U. S. A. 102(46), 16759–16764 (2005). 10.1073/pnas.0508347102 16267128 PMC1276616

[10] H. C. Thoeny, F. De Keyzer, F. Chen, Y. Ni, W. Landuyt, E. K. Verbeken, H. Bosmans, G. Marchal, and R. Hermans, “Diffusion-weighted MR imaging in monitoring the effect of a vascular targeting agent on rhabdomyosarcoma in rats,” Radiology 234(3), 756–764 (2005). 10.1148/radiol.2343031721 15734932

[11] H. C. Thoeny, F. De Keyzer, F. Chen, V. Vandecaveye, E. K. Verbeken, B. Ahmed, X. Sun, Y. Ni, H. Bosmans, R. Hermans, A. van Oosterom, G. Marchal, and W. Landuyt, “Diffusion-weighted magnetic resonance imaging allows noninvasive *in vivo* monitoring of the effects of combretastatin A-4 phosphate after repeated administration,” Neoplasia 7(8), 779–787 (2005). 10.1593/neo.04748 16207480 PMC1501887

[12] J. Setton, N. Caria, J. Romanyshyn, L. Koutcher, S. L. Wolden, M. J. Zelefsky, N. Rowan, E. J. Sherman, M. G. Fury, D. G. Pfister, R. J. Wong, J. P. Shah, D. H. Kraus, W. Shi, Z. Zhang, K. D. Schupak, D. Y. Gelblum, S. D. Rao, and N. Y. Lee, “Intensity-modulated radiotherapy in the treatment of oropharyngeal cancer: An update of the memorial Sloan-Kettering cancer center experience,” Int. J. Radiat. Oncol., Biol., Phys. 82(1), 291–298 (2012). 10.1016/j.ijrobp.2010.10.041 21167652

[13] F. F. de Arruda, D. R. Puri, J. Zhung, A. Narayana, S. Wolden, M. Hunt, H. Stambuk, D. Pfister, D. Kraus, A. Shaha, J. Shah, and N. Y. Lee, “Intensity-modulated radiation therapy for the treatment of oropharyngeal carcinoma: The memorial Sloan-Kettering cancer center experience,” Int. J. Radiat. Oncol., Biol., Phys. 64(2), 363–373 (2006). 10.1016/j.ijrobp.2005.03.006 15925451

[14] V. Vandecaveye, P. Dirix, F. De Keyzer, K. O. de Beeck, V. Vander Poorten, I. Roebben, S. Nuyts, and R. Hermans, “Predictive value of diffusion-weighted magnetic resonance imaging during chemoradiotherapy for head and neck squamous cell carcinoma,” Eur. Radiol. 20(7), 1703–1714 (2010). 10.1007/s00330-010-1734-6 20179939

[15] S. Kim, L. Loevner, H. Quon, E. Sherman, G. Weinstein, A. Kilger, and H. Poptani, “Diffusion-weighted magnetic resonance imaging for predicting and detecting early response to chemoradiation therapy of squamous cell carcinomas of the head and neck,” Clin. Cancer Res. 15(3), 986–994 (2009). 10.1158/1078-0432.CCR-08-1287 19188170 PMC2673914

[16] H. C. Thoeny, F. De Keyzer, and A. D. King, “Diffusion-weighted MR Imaging in the head and neck,” Radiology 263(1), 19–32 (2012). 10.1148/radiol.11101821 22438440

[17] M. Micco, H. A. Vargas, I. A. Burger, M. A. Kollmeier, D. A. Goldman, K. J. Park, N. R. Abu-Rustum, H. Hricak, and E. Sala, “Combined pre-treatment MRI and ^18^F-FDG PET/CT parameters as prognostic biomarkers in patients with cervical cancer,” Eur. J. Radiol. 83(7), 1169–1176 (2014). 10.1016/j.ejrad.2014.03.024 24767630

[18] Y. Liu, R. Bai, H. Sun, H. Liu, X. Zhao, and Y. Li, “Diffusion-weighted imaging in predicting and monitoring the response of uterine cervical cancer to combined chemoradiation,” Clin. Radiol. 64(11), 1067–1074 (2009). 10.1016/j.crad.2009.07.010 19822239

[19] H. Lyng, O. Haraldseth, and E. K. Rofstad, “Measurement of cell density and necrotic fraction in human melanoma xenografts by diffusion weighted magnetic resonance imaging,” Magn. Reson. Med. 43(6), 828–836 (2000). 10.1002/1522-2594(200006)43:6¡828::AID-MRM8¿3.0.CO;2-P 10861877

[20] M. Nowosielski, W. Recheis, G. Goebel, O. Guler, G. Tinkhauser, H. Kostron, M. Schocke, T. Gotwald, G. Stockhammer, and M. Hutterer, “ADC histograms predict response to anti-angiogenic therapy in patients with recurrent high-grade glioma,” Neuroradiology 53(4), 291–302 (2011). 10.1007/s00234-010-0808-0 21125399 PMC3063200

[21] P. Dirix, V. Vandecaveye, F. De Keyzer, K. Op de Beeck, V. V. Poorten, P. Delaere, E. Verbeken, R. Hermans, and S. Nuyts, “Diffusion-weighted MRI for nodal staging of head and neck squamous cell carcinoma: Impact on radiotherapy planning,” Int. J. Radiat. Oncol., Biol., Phys. 76(3), 761–766 (2010). 10.1016/j.ijrobp.2009.02.068 19540069

[22] A. D. King, F. K. Mo, K. H. Yu, D. K. Yeung, H. Zhou, K. S. Bhatia, G. M. Tse, A. C. Vlantis, J. K. Wong, and A. T. Ahuja, “Squamous cell carcinoma of the head and neck: Diffusion-weighted MR imaging for prediction and monitoring of treatment response,” Eur. Radiol. 20(9), 2213–2220 (2010). 10.1007/s00330-010-1769-8 20309553

[23] Y. Lu, J. F. Jansen, H. E. Stambuk, G. Gupta, N. Lee, M. Gonen, A. Moreira, Y. Mazaheri, S. G. Patel, J. O. Deasy, J. P. Shah, and A. Shukla-Dave, “Comparing primary tumors and metastatic nodes in head and neck cancer using intravoxel incoherent motion imaging,” J. Comput. Assisted Tomogr. 37(3), 346–352 (2013). 10.1097/RCT.0b013e318282d935 PMC3655331 23674004

